# Supplementary material for: Characterization of Entamoeba fatty acid elongases; validation as targets and provision of promising leads for new drugs against amebiasis
Source: PLoS Pathog. 2024 Aug 22;20(8):e1012435. doi: 10.1371/journal.ppat.1012435 (PMC11340893; doi:10.1371/journal.ppat.1012435)
Supplement: S1 Text — (DOCX) [file ppat.1012435.s001.docx]

**Supplementary Text**

**LC-MS/MS-based lipidomics**

Lipid extraction and LC-MS/MS for E. *histolytica* transformants were performed as described previously [1]. Lipid extraction in stable isotope metabolic labeling and drug administration experiments was performed basically as described previously [2]. Chemicals used are; ammonium acetate (AmAc) solution for high-performance LC (HPLC), ethylenediaminetetraacetic acid (EDTA), acetonitrile (ACN), methanol (MeOH), 2-propanol (IPA), and ultrapure water (H_2_O) of quadrupole time-of-flight MS (QTOF-MS) grade were purchased from FUJIFILM Wako Pure Chemical Corp. (Osaka, Japan). Methyl tert-butyl ether (MTBE) for HPLC was from Sigma-Aldrich (Tokyo, Japan). Acetic acid of LC-MS grade was from Honeywell Research Chemicals (New Jersey, US). To extract lipids, 225 μL of methanol (MeOH) was added into the cell pellets. The suspension was sonicated in BIORUPTOR II (CosmoBio, Tokyo, Japan) for 10 cycles (30 sec ON/OFF at 4 °C). After sonication, 750 μL of methyl tert-butyl ether (MTBE) was added and the suspension was mixed with a vortex mixer for 10 s. Then, 188 μL of H_2_O was added and the suspension was mixed again with a vortex mixer for 10 s. After centrifuging at 14,000 rpm for 2 min, 350 μL of the upper organic phase was collected in a clean tube and dried up by a centrifugal evaporator. The pellet was suspended in 100 μL MeOH containing 1 μL of EquiSPLASH and 1 μM FA 16:0-d3 and FA 18:0-d3.

For the stable isotope labeling experiment, an LC/QTOF-MS system consisting of an Exion LC and a ZenoTOF 7600 with an electrospray ionization ion source (SCIEX, Framingham, MA, U.S.A.) was used. Lipids were separated by the column of Unison UK-C18 MF (50 × 2.0 mm, 3 μm, Imtakt Corp., Kyoto, Japan) using two mobile phases [(A) ACN:MeOH:H_2_O (1:1:3, v/v/v) and (B) ACN:IPA (1:9, v/v)]. Both solvents contained 10 nM EDTA and 5 mM AmAc. The injection volume, flow rate, sample rack temperature, and column oven temperature were set at 1 μL, 300 μL/min, 4 °C, and 45 °C, respectively. The gradient condition used is as follows: 0.1% (B) (1 min), 0.1‒40% (B) (4 min), 40‒64% (B) (2.5 min), 64‒71% (B) (4.5 min), 71‒82.5% (B) (0.5 min), 82.5‒85% (B) (6.5 min), 85‒99.9% (B) (0.1 min), 99.9% (B) (1.4 min), 99.9‒0.1% (B) (0.1 min), and 0.1% (B) (4.4 min). A data-dependent MS/MS acquisition mode was used. The DDA method was set in both positive and negative ion modes: MS1 and MS2 mass ranges, *m/z* 75–1250; MS1 accumulation time, 200 ms; Q1 resolution, units; MS2 accumulation time, 50 ms; maximum candidate ions, 10; CAD gas, 7; intensity threshold for DDA, 10 cps; dynamic background subtraction, ticked; and no inclusion or exclusion lists were used. The following settings were used for positive/negative ion mode, independently: ion source gas 1, 40/50 psi; ion source gas 2, 80/50 psi; curtain gas, 30/35 psi; source temperature, 250/300 °C; spray voltage, 5500/-4500 V; declustering potential, 80/-80 V; and collision energy, 40/-42 ± 15 eV.

For the drug administration experiment, 1290 Bio UHPLC coupled with 6546 QTOF system (Agilent, Santa Clara, CA, USA) was used. In LC part, two mobile phases [(A) ACN:MeOH:H_2_O (1:1:3, v/v/v) and (B) ACN:IPA (1:9, v/v) with 5 mM AmAc and 0.1% acetic acid] were used, and the other conditions for LC were the same as described above. The MS settings used are as follows: gas temperature, 225 °C; gas flow, 12 L/min; nebulizer (psig), 55; sheath gas temperature, 300 °C; sheath gas flow, 11 L/min; vcap, 3500 V; nozzle voltage, 1000V; fragmentor, 175 V; skimmer 65V; octupole RF Vpp, 750 V; MS1 and MS2 ranges, *m/z* 75-1250; isolation width, narrow (~1.3 *m/z*); and collision energy, 20/30 eV. The DDA mode (set to top 7) was used for positive- and negative ion modes. The accumulation time for MS1 and MS2 were set to 200 ms and 100 ms, respectively. All raw LC-MS data were available on the RIKEN DROPMet website (http://prime.psc.riken.jp/menta.cgi/prime/drop_index) under the index number DM0056.

1. Mi-Ichi F, Ikeda K, Tsugawa H, Deloer S, Yoshida H, Arita M. Stage-Specific De Novo Synthesis of Very-Long-Chain Dihydroceramides Confers Dormancy to Entamoeba Parasites. mSphere. 2021;6(2):e0017422. Epub 2021/03/19. doi: 10.1128/mSphere.00174-21. PubMed PMID: 33731470; PubMed Central PMCID: PMCPMC8546694.

2. Tokiyoshi K, Matsuzawa Y, Takahashi M, Takeda H, Hasegawa M, Miyamoto J, et al. Using Data-Dependent and -Independent Hybrid Acquisitions for Fast Liquid Chromatography-Based Untargeted Lipidomics. Analytical chemistry. 2024;96(3):991-6. Epub 2024/01/11. doi: 10.1021/acs.analchem.3c04400. PubMed PMID: 38206184.
