# Supplementary figures and images for: Characterization of Entamoeba fatty acid elongases; validation as targets and provision of promising leads for new drugs against amebiasis
Source: PLoS Pathog. 2024 Aug 22;20(8):e1012435. doi: 10.1371/journal.ppat.1012435 (PMC11340893; doi:10.1371/journal.ppat.1012435)

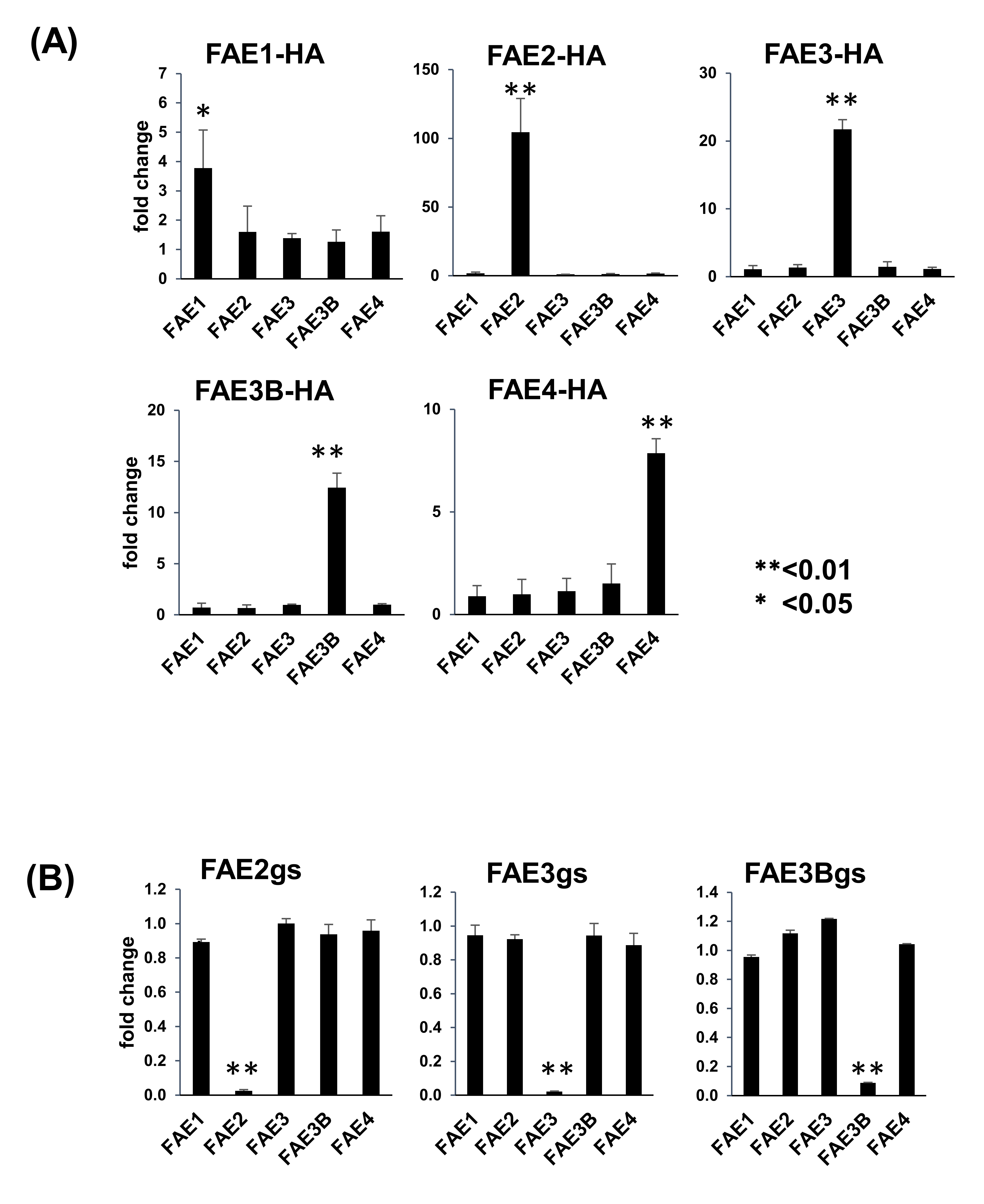

Supplement: S1 Fig — (A, B) The transcription levels of FAE genes in each transformant were quantified by quantitative reverse transcription-PCR (qRT-PCR) using the EhRNApol gene as a control. The average (bars) and standard deviation (SD) from the average (error bars) were calculated from the data obtained from duplicates of three independent experiments. *, P<0.05; **, P<0.01. (TIF) [file ppat.1012435.s002.tif]

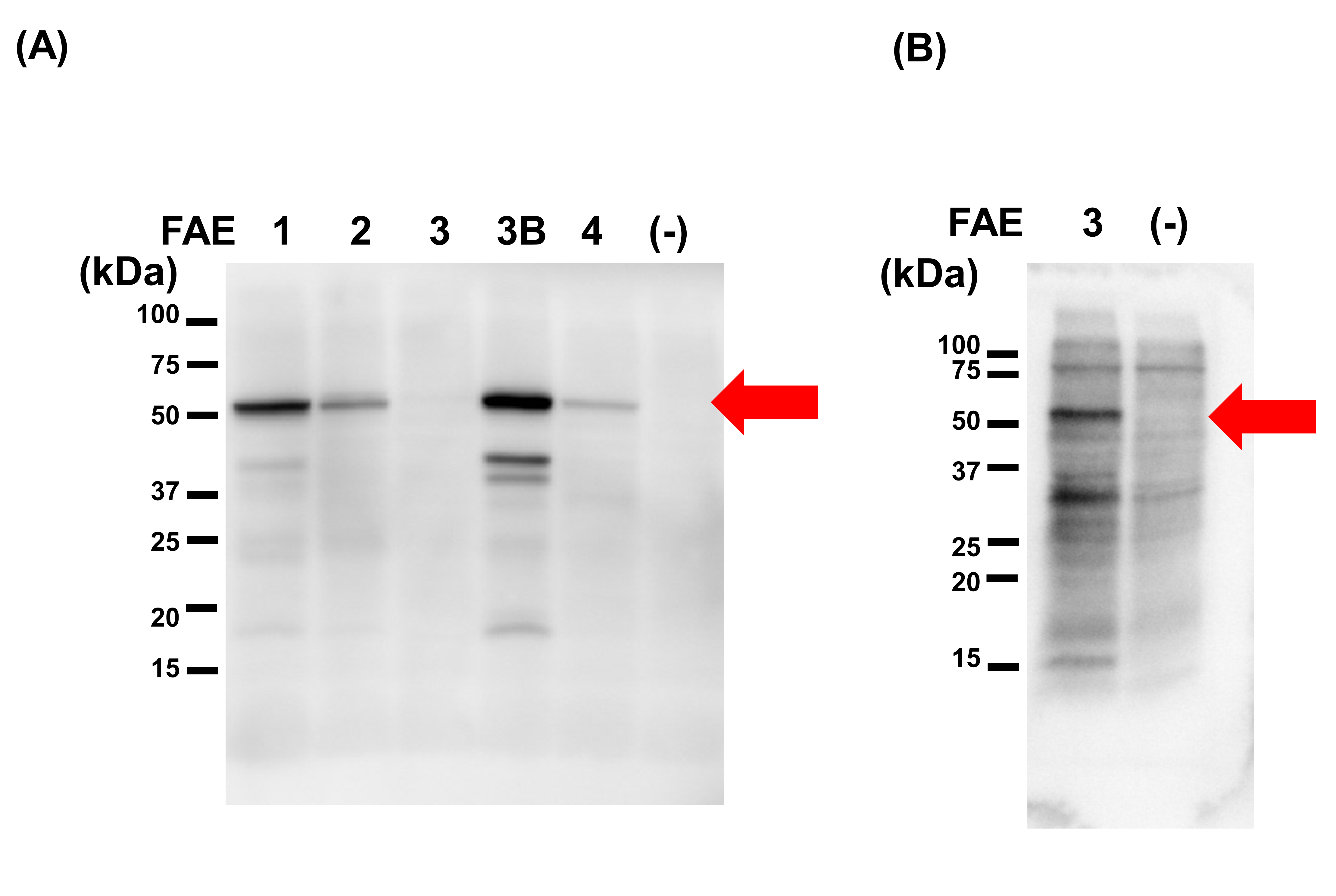

Supplement: S2 Fig — (A) All transformants and control lysates were loaded. (B) FAE3-HA and control cell lysates were only loaded to enhance the signal. (TIF) [file ppat.1012435.s003.tif]

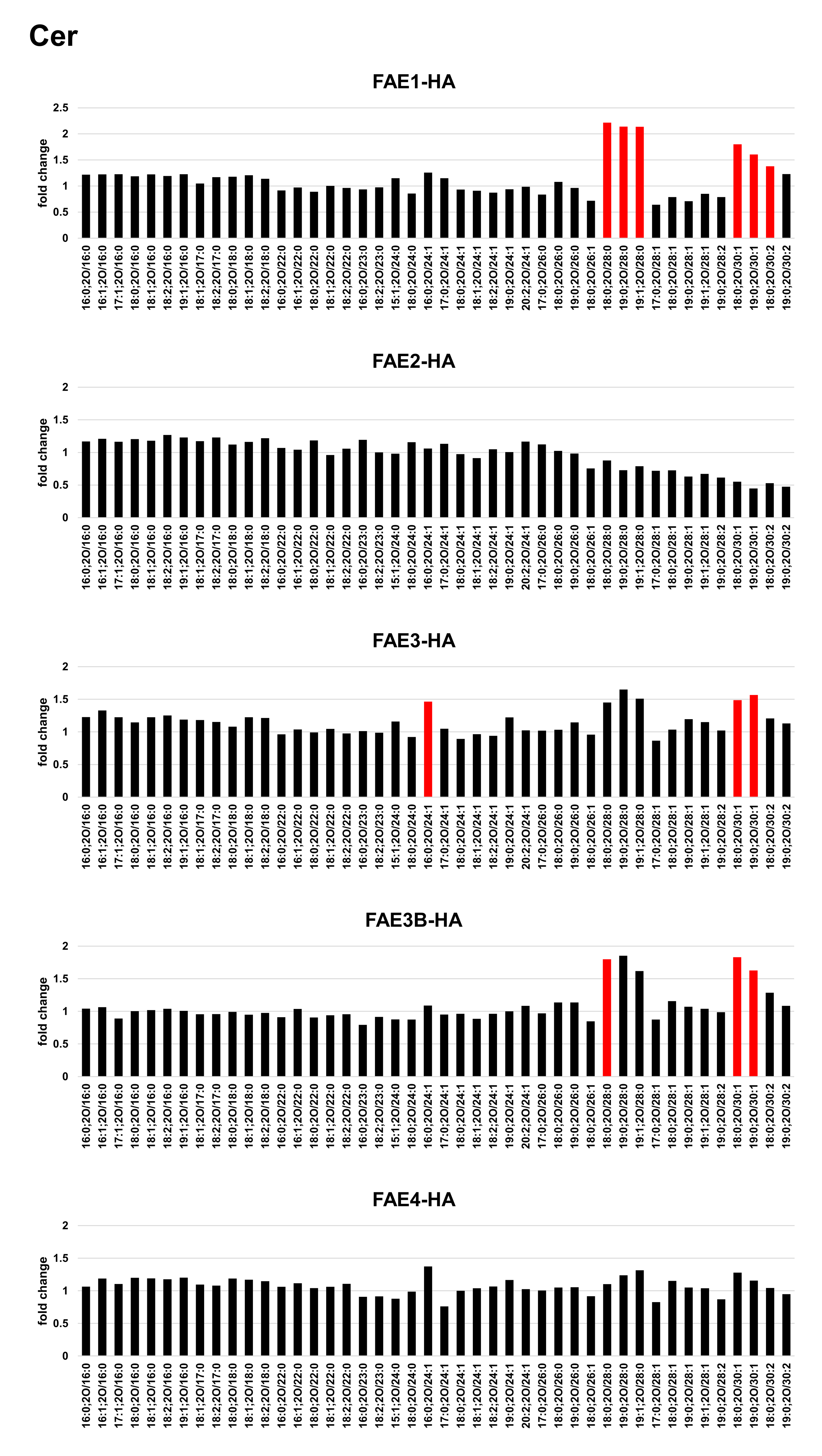

Supplement: S3 Fig — Continued from Fig 3. (TIF) [file ppat.1012435.s004.tif]

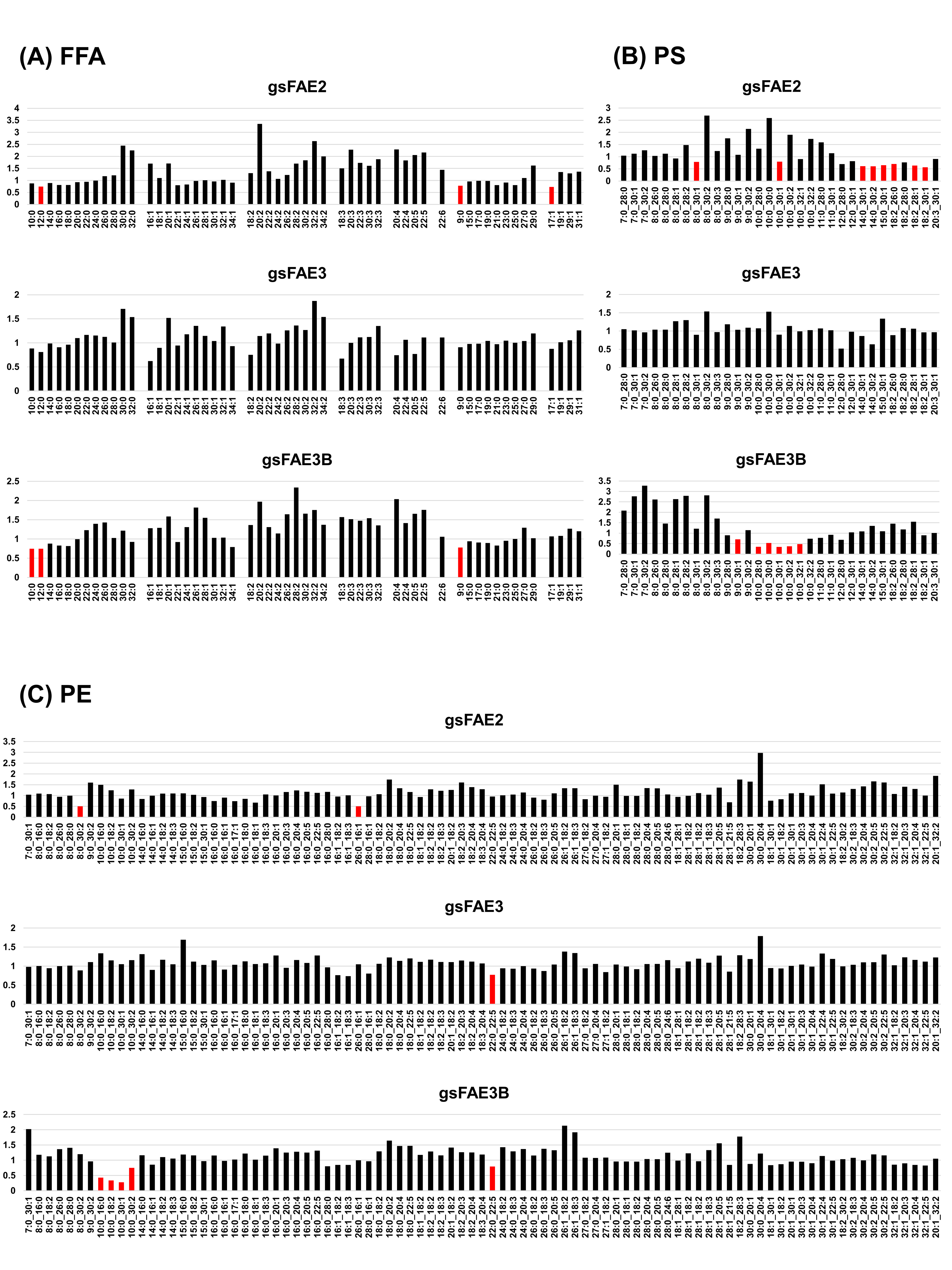

Supplement: S4 Fig — Signal intensity levels are shown as fold change to that of the control strain. Lipid species that showed a statistically significant (P<0.05) and >20% decrease are indicated by red bars. (TIF) [file ppat.1012435.s005.tif]

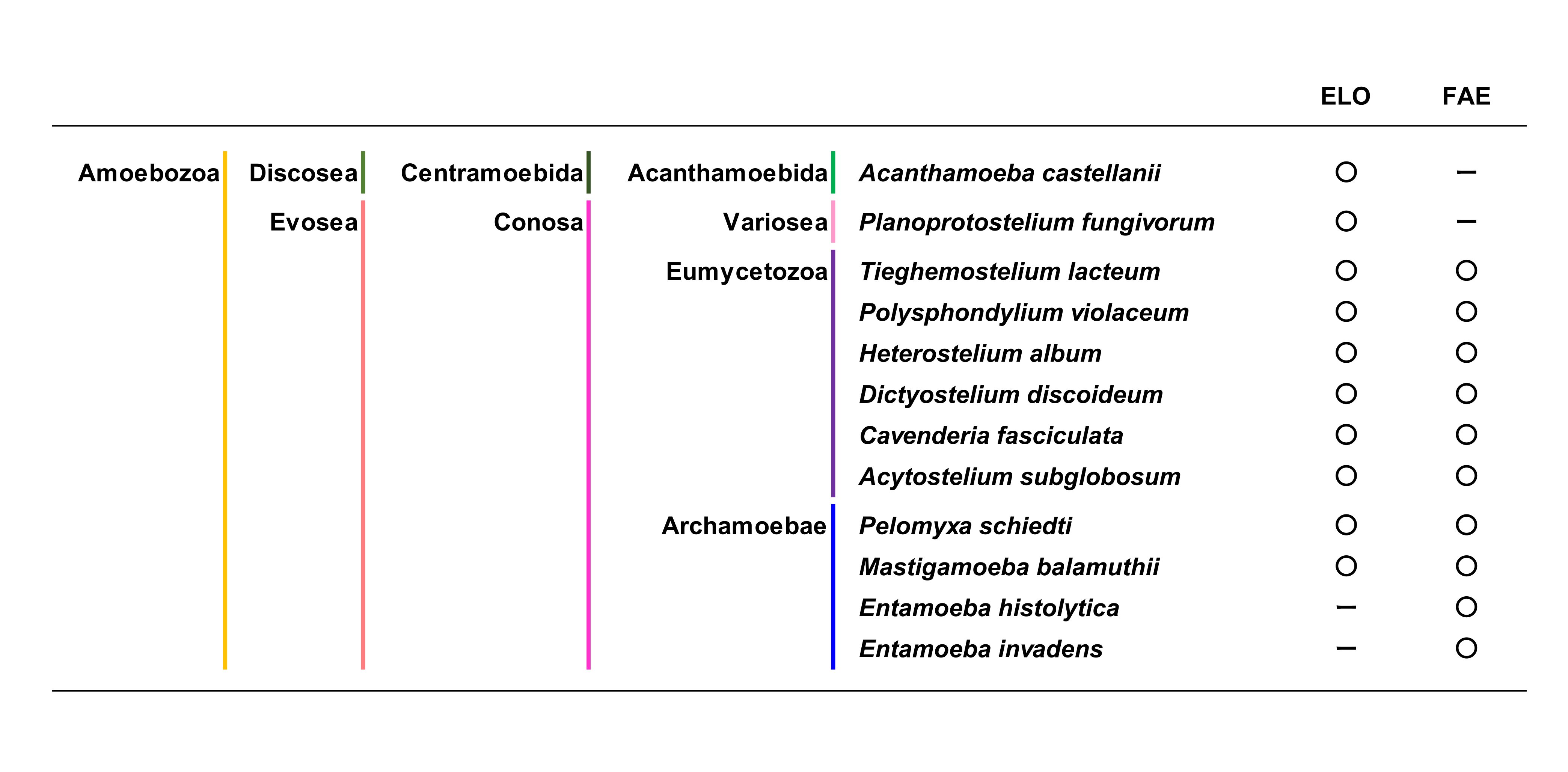

Supplement: S5 Fig — (TIF) [file ppat.1012435.s006.tif]

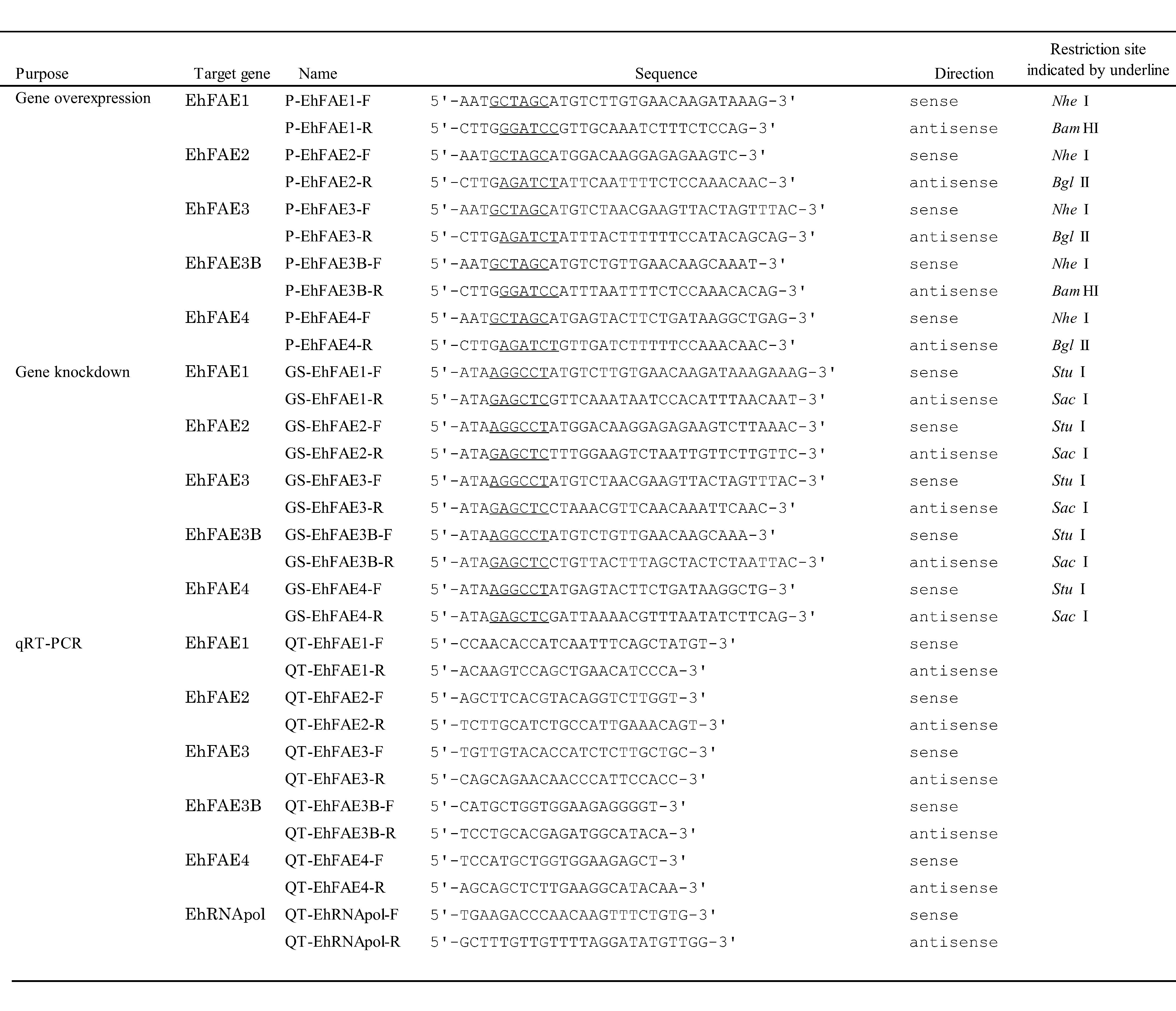

Supplement: S1 Table — (TIF) [file ppat.1012435.s007.tif]

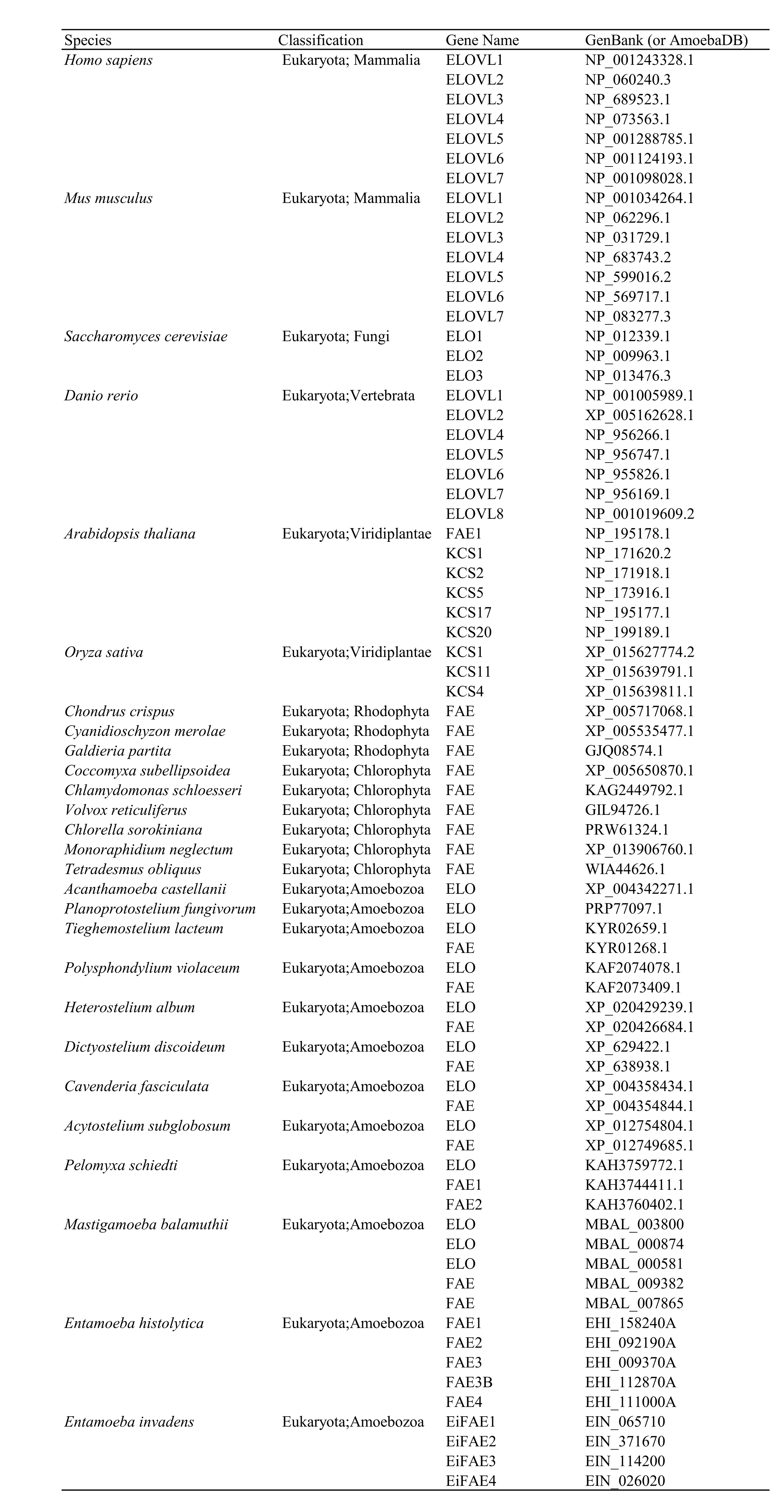

Supplement: S2 Table — GenBank accession numbers for ELOs and FAEs, which were used to infer the phylogenetic relationship shown in Fig 2A, are listed. (TIF) [file ppat.1012435.s008.tif]
