# Supplementary material for: Characterization of Entamoeba fatty acid elongases; validation as targets and provision of promising leads for new drugs against amebiasis
Source: PLoS Pathog. 2024 Aug 22;20(8):e1012435. doi: 10.1371/journal.ppat.1012435 (PMC11340893; doi:10.1371/journal.ppat.1012435)
Supplement: Attachment — Submitted filename: Point-by-point responses 240717 Fumika M.docx [file ppat.1012435.s009.docx]

**Response to comments raised by Reviewer 2.**

We greatly appreciate your thorough review of our submitted manuscript and your candid and helpful comments.

**Major Comment** **1**. The rationale behind overexpressing EhFAE genes is unclear. Are EhFAE genes expressed differentially during differentiation, exposure to various in vitro and in vivo models to study amebiasis, or under stress conditions, which could justify this approach? The authors possess ample transcriptomic data for this parasite that could be referenced to provide a physiological rationale for their decision to overexpress EhFAE genes. While the authors have demonstrated overexpression at the mRNA level, it's also crucial to confirm overexpression at the protein level through Western blotting and using HA antibodies. The authors should provide more detailed information in the Materials and Methods section regarding the concentration of G418 used to grow their transfected parasites. Specifically, it is important to clarify whether the same concentration was applied to all transfectants. This detail is crucial because the fold change in EhFAE expression varies significantly depending on the EhFAE species being overexpressed.

**Response**. The rationale for overexpressing EhFAE genes was to determine the substrate preference and the product spectra of each EhFAE isozyme in combination with untargeted lipidomics. Despite being an indirect biochemical analysis, this approach appears to be among the best to characterize the first enzymes in the fatty acid elongation system because substrate specificity can be analyzed in natural substrate conditions. The fate of products can also be traced; incorporation in glycerophospholipids and sphingolipids. We have added a brief explanation for overexpressing EhFAE genes to the relevant part of the revised manuscript [lines 158-161 in the revised version in which the changes are marked (Marked file)].

In agreement with the reviewer’s comment, we performed western blotting using an anti-HA antibody and we show the result as a new Fig. S2 in the revised manuscript. We have also added descriptions to the Materials and Methods section and to the legend for the new Fig. S2 (lines 494-502 and 218-220 in Marked file). We have also added the G418 concentration to the Materials and Methods section (lines 485-487 in Marked file).

**Major Comment** **2**. It remains unclear why the authors did not opt for a loss-of-function approach, such as RNAi-based gene silencing or other available methods for this parasite. Another notable aspect of the methodology of this manuscript is the complete absence of biochemical characterization of the various EhFAE proteins identified in this study. Furthermore, such characterization would be helpful in elucidating the mode of action of the herbicide compounds utilized in this study.

**Response**. In accordance with the reviewer’s comment, we attempted to establish transformants in which each targeted EhFAE gene was specifically knocked down. We obtained three gene-silenced transformants (EhFAE2, EhFAE3, and EhFAE3Bgs) and analyzed their lipid profiles by lipidomics. We could not subculture EhFAE1 or EhFAE 4gs under G418 selection. The results are shown as new Figs. S1B and S4 in the revised manuscript. We have added descriptions of these experiments to the Results and Material and Methods sections (lines 196-209, 211-216, 233-236, 479-489 in Marked file) .

We agree with the reviewer’s comment concerning the lack of biochemical characterization and also with the importance of biochemical characterization to determine a basis for a mode of action of the herbicides. Studies employing such an orthodox biochemical approach, which are not straightforward in this case, will be required to address this issue.

**Major Comment** **3**. The data concerning the use of herbicides to hinder the growth and differentiation of Entamoeba are intriguing. A proposed mechanism of action related to Ei suggests that cyst permeability is impaired upon herbicide treatment. However, what effects herbicide treatment may have on membrane permeability in Eh trophozoites remain to be elucidated.

**Response**. We agree with the reviewer’s comment, and actually we did analyze the effect of the three herbicides on the trophozoite cells. As the result, under blight-field microscopy, we could not observe the cells whose structures were retained. This is probably because, in contrast to cysts, trophozoites are too fragile after drug treatment to find non-lysed cells. Therefore, we could not provide mechanistic insight into the flow cytometry results of IC_50_s determination.

**Major Comment** **4**. A recent review has underscored the pivotal role of lipids in E. histolytica pathogenesis (doi: 10.3389/fcimb.2020.00075, certainly a valuable addition to the reference list of this manuscript). In my view, the manuscript would greatly benefit from in vivo insights into the virulence of the parasite when its EhFAE activities are impaired or at least from investigating the effects of herbicide treatments.

**Response**. In accordance with the reviewer’s comment, we have discussed the potential function of EhFAE isozymes in *E*. *histolytica* virulence (lines 416-424 and reference number 17 in Marked file). The review mentioned by the reviewer does indeed describe glycerophospholipids and their lyso-forms to be a part of or to appear to act as virulence factors in *E*. *histolytica*. We have also cited this review in the relevant part in the text (lines 115 and 118 in Marked file).

**Minor Comment** **1**. lane 28-29. It causes amebiasis for which clinical options are inadequate; therefore, therapeutic advances are urgently needed. This sentence is certainly overstated. Very efficient treatments are available for amebiasis, such as metronidazole (please mention current treatment in the introduction). Same remark for lane 62-63.

**Response**. In agreement with the reviewer’s comment, we have revised the text and have cited a new reference (lines 29-33 and 66-70 and references number 5 and 6 in Marked file).

Note that three new supplemental figures (Figs. S2, S3, and S4) were added; therefore, Fig. S2 in the original version has been changed to Fig. S5 in the revised manuscript.

**Response to comments raised by Reviewer 3.**

We greatly appreciate your thorough review of our submitted manuscript and your candid and helpful comments.

**Major Comment 1**. There are no statistics indicated for Fig S1 or Fig 3. Were these data analyzed for significant differences? The findings of Fig 3A-B in particular are very central to the paper’s conclusions and would benefit from significance testing.

**Response**. In agreement with the reviewer’s comment, we performed Student’s t test on the data in Figs. S1 and 3, and the results are shown in revised Figs. S1A, 3, S3, 4, and S4. We also applied the same analysis to the newly added Figs. S1B and S4 (in response to Reviewer 2’s Major comment 2). Accordingly, we have revised the original Figs. 3 and S1 and their legends and added new legends to Fig. S3 and S4. We have also discussed the results in the Discussion section and added descriptions to the Materials and Methods section [lines 365-373 and 519-527 in the revised version in which the changes are marked (Marked file)].

**Minor Comment** **1**. Line 86 appears to have a typo. I believe the word “gnomically” is meant to be “genomically”

**Response**. We have corrected it. Thank you.

**Minor Comment** **2**. Because the available information of AmoebaDB changes with every version, it is useful for reproducibility to note in manuscript methods or main text which release number of AmoebaDB was used.

**Response**. In agreement with the reviewer’s comment, we have added the release number on first mention and a brief explanation for further treatment to the revised manuscript [lines 93-94 (Marked file)]

**Minor Comment** **3**. “Cyst” is misspelled in Table 1 as “Cyt”

**Response**. We have corrected it. Thank you again.

**Minor Comment** **4**. The concentrations listed in line 242 are referred to as “~IC50s” but these values are 2-3x the IC50. Adding graphs of the full curves for IC50 experiments, would be beneficial for the reader to determine the expected inhibition at concentrations other than the IC50.

**Response**. In agreement with the reviewer’s comment, we have removed the word “~IC50s” and have added graphs showing the full curves for IC_50_ experiments as new Fig. 4B and C. We have also integrated Table 1 in the original version as Fig. 5A in the revised manuscript. Accordingly, we revised the legend for Fig. 5 and Fig. 4A, B, and C in the original manuscript have been changed to Fig. 5D, E, and F, respectively, in the revised manuscript.

**Minor Comment** **5**. It would be helpful to list the criteria for determining cyst formation within the manuscript in addition to referencing the previous methods paper. For example, the authors could specify something like “Calcofluor positive, Evan’s blue negative cells were counted as mature cysts.”

**Response**. In agreement with the reviewer’s comment, we have added a brief explanation of the characteristics of the dyes used for the flow cytometry and the criteria for assessing the effectiveness of the three herbicides. We have also cited an appropriate reference in the revised manuscript (lines 265-270 and reference number 23 in Marked file).

**Minor Comment** **6**. Do VLC Cer-NDSs have known roles in affecting membrane permeability? If so, this would strengthen the paper’s argument that VLC Cer-NDSs are the cause of encystation defects in FAE inhibitor treated samples.

**Response**. Yes, VLC Cer-NDSs are involved in generating membrane impermeability in encysting cells (Mi-ichi *et al*, ***mSphere***, 2021). Interestingly, physiological resemblance is seen between mature *Entamoeba* cysts and skin barrier function to retain water and lipids in humans and mice, with VLC Cer-NSs (ceramides) (FA portion of C24–C30) and acyl-Cer-NSs (FA portion of C30–C36) being highly accumulated (Kihara A, **Prog Lipid Res**, 2016). We have revised the relevant parts and cited a new reference (lines 321-325 and reference number 20 in Marked file).

Note that three new supplemental figures (Figs. S2, S3, and S4) were added; therefore, Fig. S2 in the original version has been changed to Fig. S5 in the revised manuscript.
